# Predicting the behavior of microfluidic circuits made from discrete elements

**DOI:** 10.1038/srep15609

**Published:** 2015-10-30

**Authors:** Krisna C. Bhargava, Bryant Thompson, Danish Iqbal, Noah Malmstadt

**Affiliations:** 1 Mork Family Department of Chemical Engineering and Materials Science, University of Southern California, Los Angeles, CA 90089; 2 Department of Biomedical Engineering, University of Southern California, Los Angeles, CA 90089.

## Abstract

Microfluidic devices can be used to execute a variety of continuous flow analytical and synthetic chemistry protocols with a great degree of precision. The growing availability of additive manufacturing has enabled the design of microfluidic devices with new functionality and complexity. However, these devices are prone to larger manufacturing variation than is typical of those made with micromachining or soft lithography. In this report, we demonstrate a design-for-manufacturing workflow that addresses performance variation at the microfluidic element and circuit level, in context of mass-manufacturing and additive manufacturing. Our approach relies on discrete microfluidic elements that are characterized by their terminal hydraulic resistance and associated tolerance. Network analysis is employed to construct simple analytical design rules for model microfluidic circuits. Monte Carlo analysis is employed at both the individual element and circuit level to establish expected performance metrics for several specific circuit configurations. A protocol based on osmometry is used to experimentally probe mixing behavior in circuits in order to validate these approaches. The overall workflow is applied to two application circuits with immediate use at on the bench-top: series and parallel mixing circuits that are modularly programmable, virtually predictable, highly precise, and operable by hand.

Additive manufacturing is rapidly becoming a viable alternative to micromachining and soft lithography for fabricating micro- and milli-fluidic devices^1^,^2^,^3^,^4^,^5^. Methods such as stereolithography (SLA) or extrusion-based processes (*e.g.* fused-deposition modeling, or FDM) enable entire devices with non-planar channel geometries to be fabricated rapidly and with less resources relative to traditional methods^6^. However, additive manufacturing is generally less precise than micromachining, leading to the possibility of performance errors in microfluidic systems designed to precisely control fluid transport and mixing. The impact of manufacturing variability on microfluidic circuit functions has not been explored quantitatively in the literature; errors in concentration of flows in complex microfluidic networks are generally unpredictable and must be addressed on a case-by-case, *ad hoc* basis. Additive manufacturing makes this sort of quantitative analysis possible by introducing a standardized fabrication technology as well as by encoding microfluidic system designs as digital, machine-interpreted files.

Previously, we introduced a platform of self-aligned discrete microfluidic elements manufactured using SLA that are reversibly connectable and described by their terminal flow characteristics much like discrete elements in electronic systems^7^. This system lends itself to the construction of reconfigurable, modular, three-dimensionally complex, and hierarchically designed microfluidic devices from a library of standardized components suitable for mass manufacturing. In this work, we develop this system further by demonstrating a virtual implementation strategy and experimental probing procedure that address predicting variations in performance. This strategy has three parts: (A) definition of a component library of passive elements qualified by their expected variation due to manufacturing, (B) network analysis to derive mixing operability of some simple microfluidic circuits with useful application on the bench-top, and (C) prediction of network performance variation using statistical analysis methods.

In (A), we develop an element library that is intuitively compatible with linear circuit analysis and accompanying statistical analysis techniques; hydraulic resistance values of each element were selected with convenience for designers in mind. Channel geometry was then deduced in order to yield these well-defined values of hydraulic resistance. In (B), microfluidic circuit topologies for source-invariant parallel and series mixing were conceived of and characterized by simple mathematical rules as a model system for network analysis. Network analysis is a powerful method for gaining insight into the operation of monolithic microfluidic circuits (see^8^,^9^,^10^,^11^,^12^,^13^ for a diverse set of examples), but in general has not been used as a central tool for design. In (C), a complete virtual implementation of each microfluidic circuit was devised, including expected manufacturing variation in components, in order to simulate the realistic scenarios for network performance. This was accomplished through applying mechanistic understanding of stereolithography to the statistical calculation of hydraulic resistance tolerances at the module level in (A), and further applying understanding of module level tolerances to the network analysis performed in (B). Ultimately, experimental realizations of these mixing circuits were assembled and probed using osmotic solutions in order to validate these models.

## Application Circuits

A fundamental process in analytical and synthetic chemistry is the mixing of small quantities of fluids. Typically, this is accomplished using syringes, pipettes, burettes, and a number of other glass and plastic tools that are operated by hand to manually withdraw fluids from source containers and procedurally deposit them into a sample container. The error in the final composition of a mixture is largely attributed to error in operating these tools, or “source-variant”, and can have serious consequences on the repeatability of procedures critical to research and clinical activities. Consequently, electronically controlled robotic systems that automate these procedural tasks have been developed in order alleviate operator-induced error, create better consistency in the physical operation of instruments, and speed up processing of samples overall. These systems are often prohibitively expensive, carry a large learning curve, and require significant infrastructural support. There is therefore a need for hand-held tools for precise, predictable mixing of low volume solutions that are insensitive to typical operator and instrument errors, or “source-invariant”. The model circuits presented in this work are designed to serve this practical need, leveraging the reconfigurable and resistance-centric aspects of our overall discrete element system, while borrowing inspiration from the large volume of microfluidic mixing circuit design literature available^14^,^15^,^16^,^17^. Microfluidic technologies for controlled mixing and dilution demonstrated in the literature thus far have been focused on the generation of concentration gradients that mimic biologically relevant fluidic environments. Devices capable of performing parallel and serial mixing^18^ multi-layer dilutions^19^ and logarithmic concentration gradients^20^ have provided microfluidic chip designers with several strategies for manipulating the concentration of solutions. While these methods represent a substantial effort to construct application-specific microfluidic circuits, few of these techniques make it simple for designers to tune mixing factors in a modular sense.

## Results and Discussion

### Microfluidic Resistor Element Library

**Figure 1:**
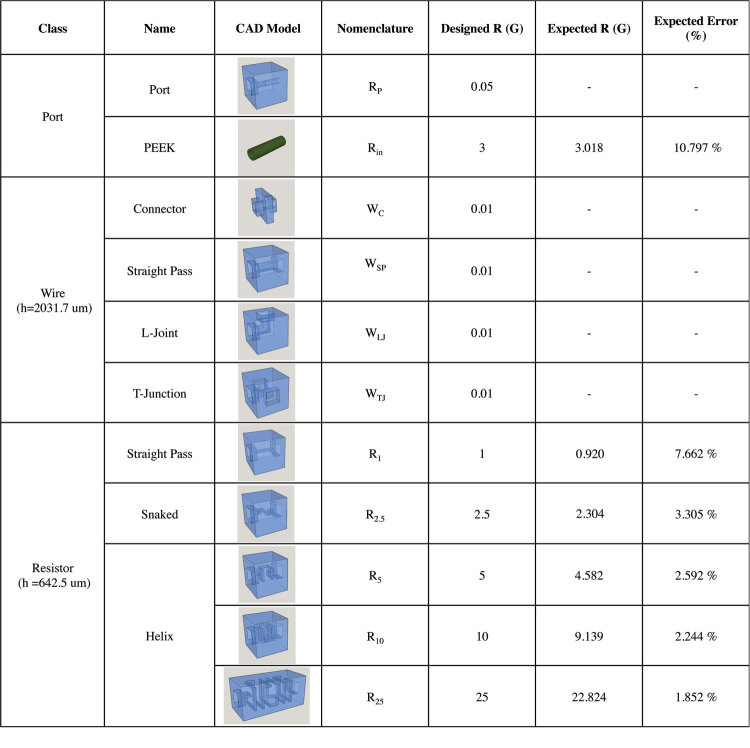
Library of constructed discrete elements organized according to their designed hydraulic resistance, with units denoted as G, short for GPa-s-m^−3^. Resistor class components were designed with a 642.5 μm channel side-length, allowing resistance to be manipulated by channel length that is achieved by snaking and coiling channels within individual resistance components. Expected Resistance and Error (2σ) determined through Monte Carlo Analysis from measured channel side length, which statistically ensures that 95% of designed components will fall within the stated resistance tolerance.

Laminar microscale flows can be analyzed in terms of their hydraulic resistance as determined by channel size and morphology^8^. In other words, it is possible to develop a set of fluidic elements with varying channel size that are defined on the basis of total hydraulic resistance per component. We therefore developed a library of microfluidic channel elements categorized by their hydraulic resistance in a manner that allows designers to rapidly prototype designs on paper (Fig. 1). Channel sizes were restricted such that the Reynolds Number of each element was strictly indicative of laminar flow for rates as high as 200 mL/hr. The community practices set by manufacturers of standardized discrete electronic resistors was adopted, wherein a typical functional unit of resistance is two to three orders of magnitude larger than that of the so-called “parasitic” resistance in a wire.

Consider the hydraulic resistance of a segment of straight channel with square cross-section (1)^8^:


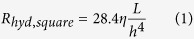


Here, the length of a channel segment is given by *L*, the height or width of the cross-section is *h*, and the dynamic viscosity of the conducted fluid is *η*. For the purposes of this study, the definition of such elements is restricted to the flow of pure water, at 20 °C, the temperature of our experiments. Therefore, a value of 1 mPa · s was used for *η* throughout. We define a unit of hydraulic resistance “G” as short-hand notation for 1 *GPa-s-m*^–3^. This corresponds to the resistance of a reference channel element of 6 mm length and 642.5 μm square cross-sectional height. This channel height was selected for its convenience in creating a system of resistors with values on a standard scale of integers or simple fractions. This approach was borrowed from a similar practice in discrete electronic components, wherein manufacturers primarily offer electronic resistors with values that allow for easy combination and selection by designers during the drafting of circuit schematics.

A variety of hydraulic resistor elements were constructed based on the reference 642.5 μm process height. Resistors larger than 1G were realized by snaking and coiling longer track lengths into a standardized cubic element footprint. A special class of wire-like components characterized by a 0.01 G parasitic resistance were developed by increasing the cross-section side length to 2.0317 mm such that flow conditions are still laminar at reasonably high flow rates (*e.g. Re* ~ 0.1 at 200 ml/h in the standard 6 mm reference length). Port elements for interfacing to circuits were constructed with a special height of 1.1425 mm, resulting in a parasitic resistance of 0.05 G. These stray millifluidic resistances are sufficiently less than resistor class microfluidic components such that they need not be considered in microfluidic network analysis during the design phase.

### Determination of Resistor Tolerance from Statistical Methods

The error in resistance for each element was determined from the errors associated with the stereolithographic manufacturing process. Consider the hydraulic resistance of a channel segment of rectangular cross-section height *h*, width *w*, and length *L* given in (2) and derived by solving the Navier-Stokes equation using a Fourier Series method^8^:


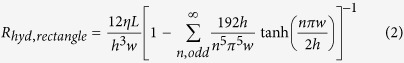


**Figure 2:**
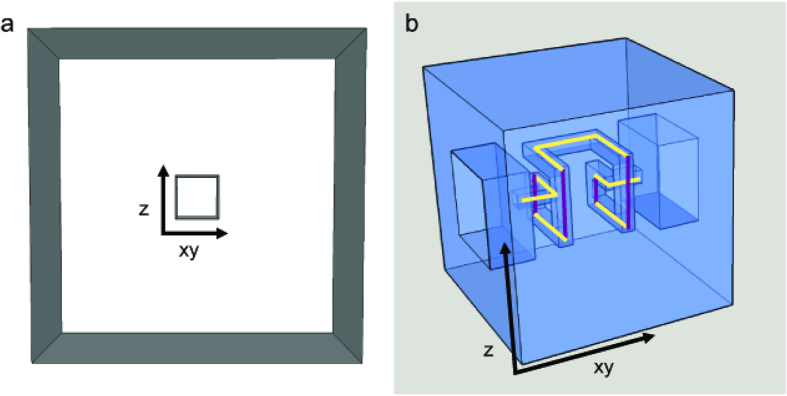
(**a**) Port opening with centered channel for a resistance component with 642.5 μm cross-sectional side length showing the precision in the print plane, xy, and the precision in the print axis, z. Due to the control mechanisms of stereolithography, precision in the xy and z plane are expected to be different. (**b**) Total component resistance was approximated by determining and adding together the resistance of segments respective to the xy-planes (yellow lines) and z-plane (purple lines). Both planes carry a particular fabrication error, determined by optically measuring the cross-sectional side length of components and creating distributions of precisions in both orientations (see Supplementary Figure 1).

The layer-by-layer stereolithographic printing method affects manufacturing tolerances/errors Δ*w*, Δ*L*, and Δ*h* due to the direction in which a channel segment is arranged with respect to the printing process (Fig. 2). This implies that the error in Δ*xy* due to the printing optics and related control mechanism that affects the so-called “xy print plane” will be different than the error Δ*z* due to the mechanism that controls the addition of photoresin layers along the “z print axis”. This results in channels that are imperfectly rectangular despite their square design. In addition, the solidification of material during printing and in post-processing may cause further anisotropic deformation of the channel. These effects appear to be secondary to those resulting from controls mechanisms in the printer, but the characterization procedure described here is translatable across variety of additive manufacturing processes and materials beyond those demonstrated experimentally. Experimental values for the print plane and print axis tolerances were determined by constructing a large number of library components with a model material (see Materials and Methods) and optically measuring their geometric cross sections (Supplementary Figure 1). These tolerances were used in a Monte Carlo simulation to predict the standard deviation in hydraulic resistance of each element in the component library (see Materials and Methods Section). Briefly, the resistance of each channel segment constituting a given element was computed using (2) with parameters *w*, *L*, and *h* drawn from pseudorandom normal distributions set by the tolerances Δ*xy* and Δ*z* depending on the orientation of that segment (Fig. 2b). The segment resistances were added in series such that final resistance for the element was computed for that particular set of draws. Ultimately, the “manufacturer’s tolerance”, or 2σ, for 5000 draws was determined such that 95% of constructed resistor elements will fall within the specified range of values (Fig. 1).

### Circuit Topologies for Parallel and Series Mixing

**Figure 3:**
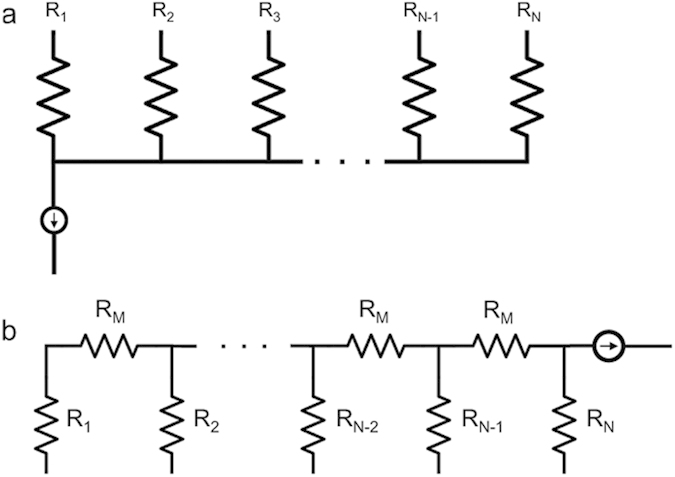
(**a**) Generalized *Fork* topology where each branch resistance experiences the same pressure drop across and mixing occurs in parallel between inputs. (**b**) Generalized *Ladder* topology where mixing occurs in a serial manner, such that branch R_1_ mixes with branch R_2_, which then passes through a mixing resistance, R_M_, and further mixes with the next adjacent branch, until branch R_N_ is reached.

This study considers two microfluidic circuit topologies, denoted as *Fork* (Fig. 3a) and *Ladder* (Fig. 3b), respectively capable of parallel and series mixing. Reagents are pulled through inlet branches by a negative flow-rate source, such that they combine at a common junction to yield the target mixture. Borrowing from the hydraulic analogy to electrical circuits^8^, the Fork topology employs the principles of parallel resistances, wherein the pressure drop seen across each input branch is equivalent. In turn, the flow rate developed across each branch is due solely to the selection of branch resistances. This allows for a system where the resulting volume fraction of each inlet fluid at the outlet is invariant to the flow rate. The Ladder topology enjoys similar independence from source variation, but represents an alternate scenario in which inlet reagents are mixed serially. Flow through the branch furthest from the source is mixed with the next adjacent branch, which is then mixed with the next adjacent branch, so on and so forth until the final mixture is created at the outlet. In other words, the Ladder topology can be thought of as recursively connected Fork topologies.

### Network Analysis and Characterization of Performance from Statistical Methods

We employed nodal analysis to study the operation of 2- and 3-inlet Fork circuits, as well as a 3-inlet Ladder circuit (Supplementary Notes 1–6). The design objective was stated in terms of *χ*, the volume fraction of a given inlet fluid in the outlet mixture. Design rules for *χ* for each inlet were in turn derived, showing how branch resistances can be selected in order to tune operation to deliver a given mixture of each feed fluid (Table 1). Note that the source-invariance of each configuration is apparent in each mixing rule: *χ* only depends on the choice of resistor, and not the withdrawal flow rate. Each circuit was then constructed using a variety of resistor combinations (Table 2a–c) and tested by running a stock NaCl solution through a single branch of each topology (see Materials and Methods Section). The resulting dilutions of the stock solution were tested using osmometry, for which the stock solution was well suited to provide results with variance far below that of the predicted manufacturing variance in network operation.

A feature of board-level electronic circuit design that enables efficient and repeatable design and manufacturing is the use of statistical methods to predict circuit behavior due to error in terminal characteristics at the element level. Similarly, the tolerance of hydraulic resistors becomes important for predicting the operation of microfluidic circuits assembled from mass-manufactured standardized elements. While simple error analysis is sufficient for most circuits involving few nodes and input reagents, complex networks are hard to analyze by hand. This analysis can be automated using numerical techniques. We extended our use of Monte Carlo analysis at the element level to simulate expected performance at the circuit level. More specifically, we generated simulated bins of pseudorandom normally distributed discrete microfluidic resistors using the tolerances derived in Fig. 1, and computed the distribution of possible outlet volume fractions using the rules in Table 1. The resulting volume fractions were analyzed for the inlet of the first branch (Branch R_1_) of each circuit topology, which was fed by the stock NaCl solution.

### Experimental Validation

**Figure 4:**
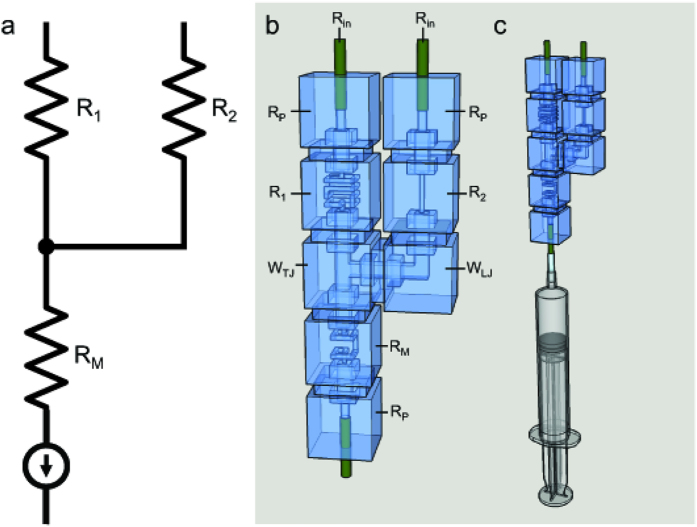
(**a**) Circuit diagram of a 2-input, 1-output fork topology where R_1_ and R_2_ are selectively chosen for desired operational output. (**b**) Equivalent hydraulic circuit where only R_1_ and R_2_, the selected resistance components reasonably contribute to mixing ratio and wire elements are construed as “parasitic”, or negligible. Note that the current source symbol represents a syringe withdrawing solutions through the inlets to the outlet. (**c**) Equivalent hydraulic circuit with syringe attached to output end to demonstrate experimental setup.

**Figure 5:**
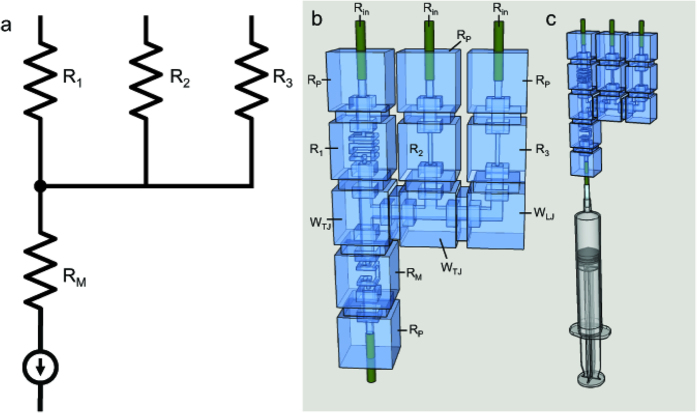
(**a**) Circuit diagram of a 3-input, 1-output fork topology where R_1_, R_2_, and R_3_ are selectively chosen for desired operational output. (**b**) Equivalent hydraulic circuit where only R_1_, R_2_, and R_3_ the selected resistance components reasonably contribute to mixing ratio and wire elements are construed as “parasitic”, or negligible. Note that the current source symbol represents a syringe withdrawing solutions through the inlets to the outlet. (**c)** Equivalent hydraulic circuit with syringe attached to output end to demonstrate experimental setup.

**Figure 6:**
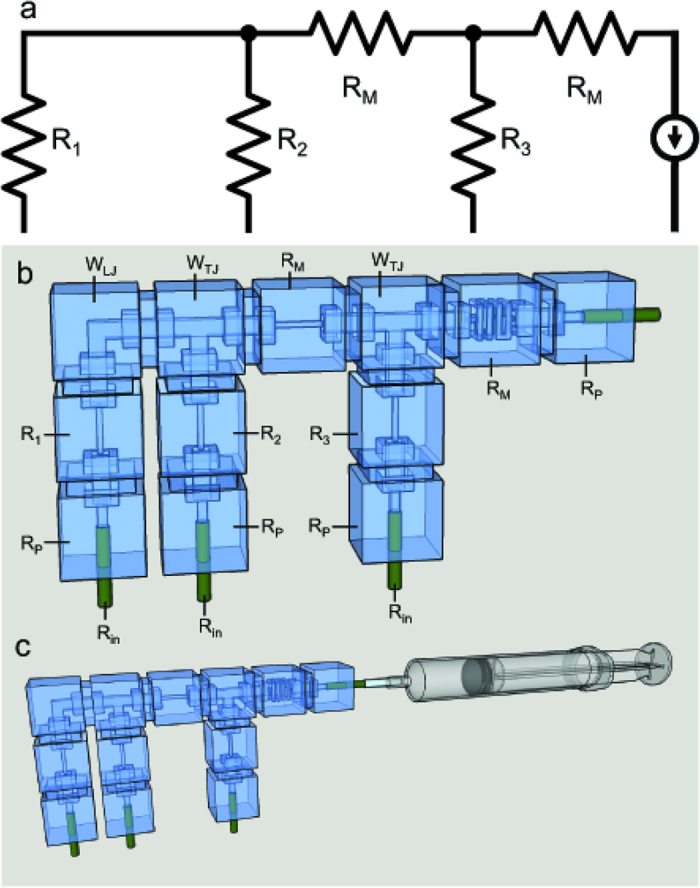
(**a**) Circuit diagram of a 3-input, 1-output ladder topology where R_1_, R_2_ and R_3_ are selectively chosen for desired operational output. (**b**) Equivalent hydraulic circuit where only R_1_, R_2_, and R_3_ the selected resistance components reasonably contribute to mixing ratio and wire elements are construed as “parasitic”, or negligible. Note that the current source symbol represents a syringe withdrawing solutions through the inlets to the outlet. (**c**) Equivalent hydraulic circuit with syringe attached to output end to demonstrate experimental setup.

**Figure 7:**
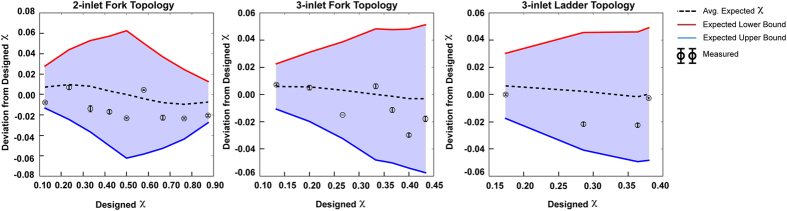
Comparison of experimental mixing ratio deviation from designed mixing ratio in comparison to simulated mixing ratio deviation to designed mixing ratio. For each graph, the upper and lower bound describe a 2σ deviation from the expected mixing ratio, such that the shaded region, effectively the simulated operating space, is established by the manufacturer tolerance that suggests 95% of the constructed resistor elements will fall within specification. Experimental data lie within the simulated operational working space for the (**a**) 2-inlet Fork Topology, (**b**) 3-inlet Fork Topology, and (**c**) 3-inlet Ladder Topology.

The 2,3-inlet Fork and 3-inlet Ladder circuits were assembled (Figs 4, 5, 6) from the library of modular microfluidic devices (Fig. 1) with the configurations given in Table 2. The volume fraction of inlet reagents was validated for each configuration by introducing a “probe” solution of measurable concentration to a single inlet, which is then mixed with diluent from remaining inlets in the circuit, and the outlet concentration is finally measured. Each circuit was constructed including an inlet resistance determined by 24.4 mm PEEK tubing (OD 1/16”). The 0.34 M NaCl probe solution was run through the branch denoted as R_1_ in each circuit and all other inlets were fed by Milli-Q water. After channels were primed by manual withdrawal, the syringe barrel was interchanged with a clean barrel to collect roughly 0.5–1 mL of resultant mixture. Osmolality of the diluted NaCl solution product was measured (see Materials and Methods), allowing for determination the volume fraction for each resistance combination, for all topologies, for three syringe barrel exchanges (effectively three repetitions). Note that the measured output volume fractions (as determined by osmometry to find NaCl concentration in the output) fall within the predicted range of variation from as-designed resistance values. In Fig. 7, simulated and experimental volume fraction data are arranged to show the deviation from the designed volume fraction, whose values for each dilution was calculated by applying the resistor combinations of Table 2 to the mixing laws presented in Table 1, for the R_1_ branch of each topology. Note that many of the experimental values for *χ* were found to be greater than those designed, resulting in a tendency for negative deviations. This seems to occur largely because the mean expected resistance for many elements is less than their designed value, reflected by an overall higher mean value for Δ*xy* and Δ*z* tolerance distribution (or *w*, *L*, and *h* channel parameters).

This study effectively demonstrated that performance variations in microfluidic systems constructed using discrete elements could be statistically predicted in context of mass manufacturing. Device processing and network assembly level error propagation was simulated using empirically determined process parameters and mechanistic understanding of the SLA process. Figure 7 shows that the measurable performance of real circuits for parallel and serial mixing constructed from a sample component library reliably perform within virtually determined bounds of precision. These circuits were useful as a modular, adjustable, and handheld laboratory tool for creating mixtures with very high levels of precision. Furthermore, the circuit topologies explored in this work are expandable to an arbitrary number of input solutions; the network and Monte Carlo analysis techniques presented here reliably enable the determination of performance of similar systems with scaled complexity.

## Materials and Methods

Each circuit topology was tested manually by feeding the R_1_ branch of each circuit with a 0.34 M NaCl probe solution and running Milli-Q water through the remaining circuit branches. A syringe was connected at the output end and was manually retracted to prime the circuit branches with their respective solutions. After priming all branches, a clean syringe replaced the priming syringe and about 1 mL of diluted mixture was collected. In order to calculate the experimental volume fraction, an osmometer (Gonotec Osmomat 3000) was used to measure the osmolality of 0.5 mL of the mixed product. The stock NaCl solution had a measured osmolarity of 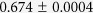 *osmol-kg*^–1^; a linear relationship between osmolarity and salt concentration was used to find the concentrations of the diluted mixtures. All components were fabricated through a contract manufacturer Fineline Prototyping (Protolabs Inc.) in Watershed 11122 XC photoresin material.

The Monte Carlo simulation was written in Python 3.4.2 using the Anaconda SciPy framework. Supplementary Figure 2 shows an overview of the procedure. Process data were collected (Supplementary Figure 1) and fit to a normal distribution, from which the standard deviation and mean values were used to find expected mean and tolerance values for all members of the resistor component library. A loop with a maximum count of 5000 tries was constructed, wherein a normally distributed pseudorandom number generator was called to create a kit of virtual resistors set by the expected values in the component library in the previous step. The kit was in turn used to calculate expected values for mixing laws, yielding a predicted *χ* value. The resulting *χ* values were compared to those expected from resistors with no manufacturing error, yielding the deviation from intended behavior expected from mass-manufactured parts.

## Additional Information

**How to cite this article**: Bhargava, K. C. *et al.* Predicting the behavior of microfluidic circuits made from discrete elements. *Sci. Rep.* **5**, 15609; doi: 10.1038/srep15609 (2015).

## Supplementary Material

Supplementary Information

## Figures and Tables

**Table 1:** General mixing rules for the 2-and 3-inlet fork microfluidic circuit topology, as well as the 3–1 ladder topology.

Circuit Topology	Mixing Law
2-Inlet Fork Mixer	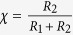
3-Inlet Fork Mixer	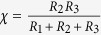
3-Inlet Ladder Mixer	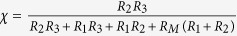

The dilution ratio, *χ*, is designed by selecting microfluidic resistors R_i_ (i = 1, 2, 3, M) from a component library such as presented in Fig. 1, and constructed in relation to the appropriate circuit model for that topology (Figs 4, 5, 6).

**Table 2:** 

a. 2-Inlet Fork Mixer					
R_1_ (G)	R_2_ (G)	R_3_ (G)	Designed χ	Expected χ	Expected Error (%)
R_1_	R_1_	–	0.500	0.499	11.859%
R_1_	R_2.5_	–	0.421	0.426	11.998%
R_1_	R_5_	–	0.333	0.341	12.511%
R_1_	R_10_	–	0.235	0.245	13.327%
R_1_	R_25_	–	0.125	0.132	14.400%
R_2.5_	R_1_	–	0.579	0.575	8.711%
R_5_	R_1_	–	0.667	0.659	6.431%
R_10_	R_1_	–	0.75	0.756	4.318%
R_25_	R_1_	–	0.875	0.868	2.247%
**b. 3-Inlet Fork Mixer**					
R_1_	R_1_	R_1_	0.333	0.333	13.587%
R_1_	R_5_	R_1_	0.200	0.206	11.569%
R_5_	R_1_	R_1_	0.400	0.397	12.169%
R_1_	R_10_	R_1_	0.133	0.139	11.418%
R_10_	R_1_	R_1_	0.433	0.430	11.930%
R_1_	R_2.5_	R_1_	0.366	0.365	12.636%
R_2.5_	R_1_	R_1_	0.266	0.270	12.754%
**c. 3-Inlet Ladder Mixer**					
R_1_	R_1_	R_1_	0.286	0.289	14.241%
R_1_	R_1_	R_5_	0.174	0.181	12.618%
R_1_	R_10_	R_1_	0.380	0.380	12.253%
R_5_	R_1_	R_1_	0.364	0.362	12.371%

Resistor Combination tables for (**a**) 2-inlet fork topology, (**b**) 3-inlet fork topology, and (**c**) 3-inlet ladder topology, where the R_1_ branch runs a 0.34 M solution, and remaining branches run Milli-Q water, which are mixed and manually withdrawn at the output end of each topology. The designed mixing ratio that utilizes as-designed resistance values, Designed χ, and expected mixing ratio from Monte Carlo simulation that takes into account build error, Expected χ, are calculated by using the respective mixing law shown in Table 1. The expected error is two standard deviations from the expected mixing ratio.
