# Supplementary material for: Predicting the behavior of microfluidic circuits made from discrete elements
Source: Sci Rep. 2015 Oct 30;5:15609. doi: 10.1038/srep15609 (PMC4626777; doi:10.1038/srep15609)
Supplement: Supplementary Information [file srep15609-s1.pdf]

SUPPLEMENTARY INFORMATION FOR:

PREDICTING THE BEHAVIOR OF MICROFLUIDIC CIRCUITS MADE FROM DISCRETE ELEMENTS

Krisna C. Bhargava, Bryant Thompson, Danish Iqbal, Noah Malmstadt

Supplementary Figure 1

Distribution of measured cross-sectional lengths in (a) xy and (b) z orientation for resistance components of 642.5-um cross-sectional channel side length. Optical micrographs determined Δxy and Δz to be 659 ± 12.47 um and 642 ± 4.24 um respectively, for 72 measurements in each direction.

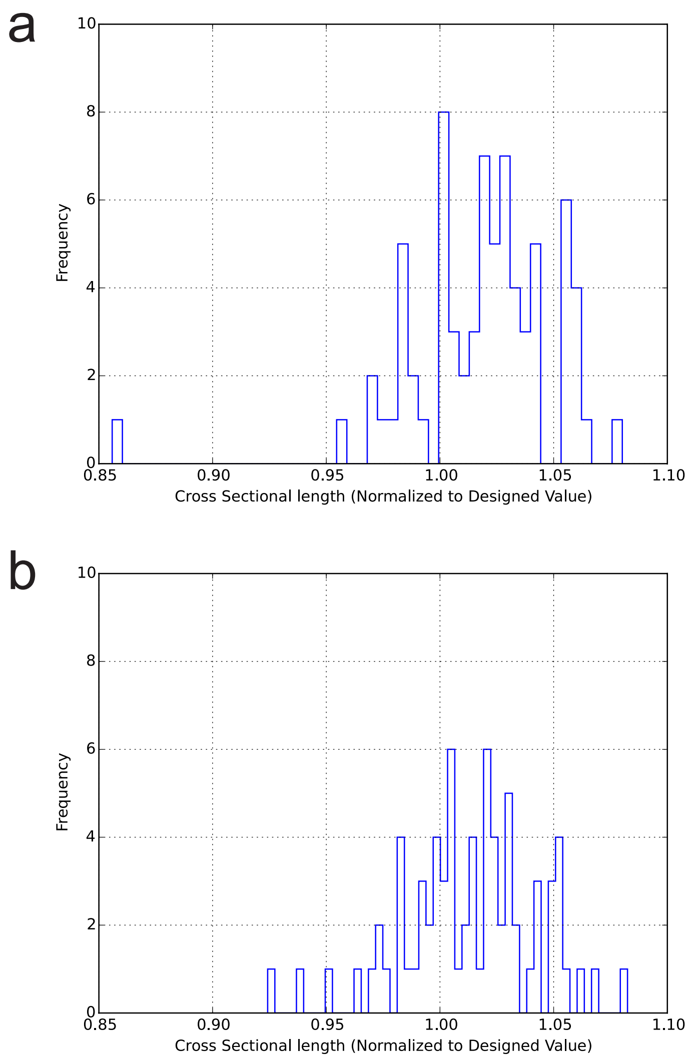

Supplementary Figure 2

Flow of control for Monte Carlo Analysis. Process data informed by SLA manufacturing methods was used to derive realistic error and mean hydraulic resistances for components used in this study. 10,000 resistor kits were then generated for a given circuit topology, and the performance extrema were derived.

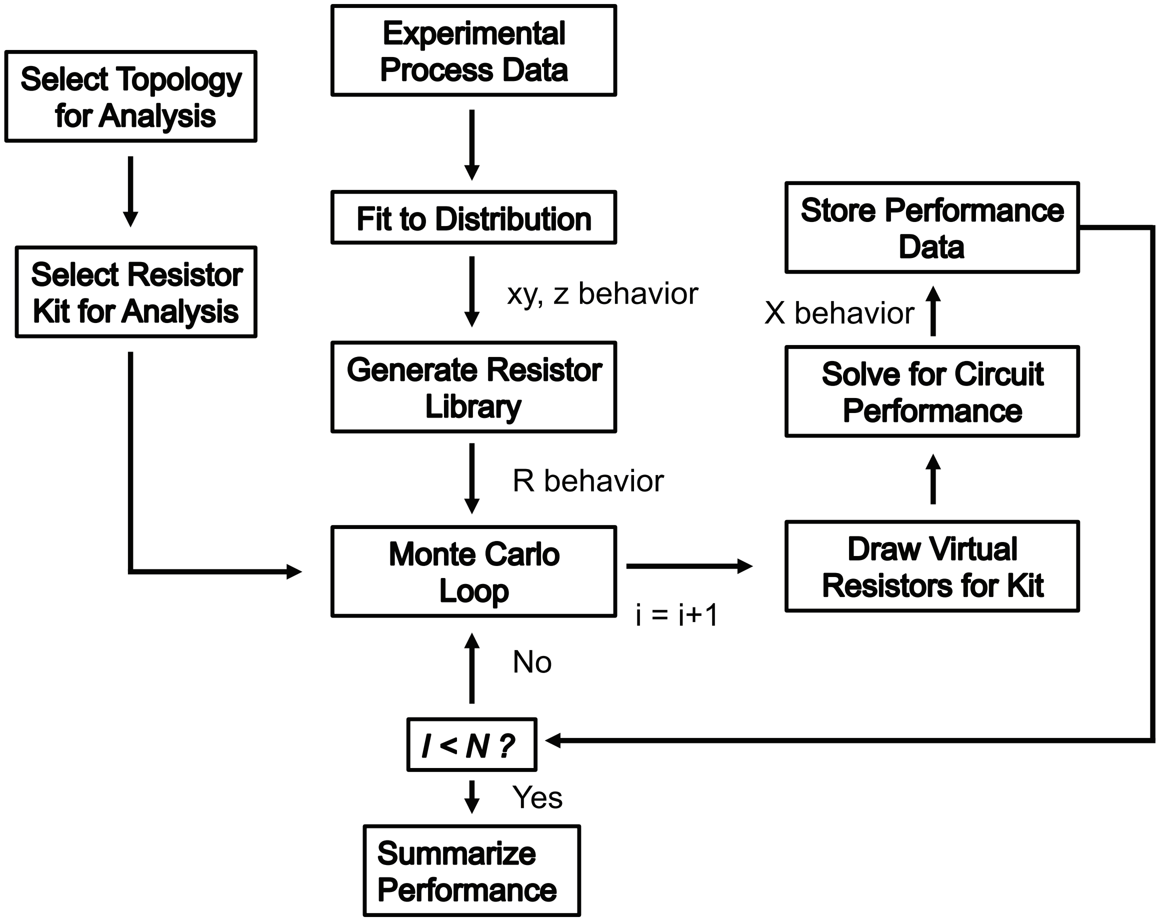

Supplementary Note 1

Analysis of 2-inlet 1-output Fork Circuit Topology

In electronic circuit theory, circuit subassemblies are often characterized by a simple set of mathematical rules that relate input and output signal information, treating the circuit as a black box. Here we consider the concentration to be relevant 'signal information' in a microfluidic circuit subassembly. Specifically, we relate choices in hydraulic resistors to inlet and outlet concentrations. These rules are then validated experimentally by probing a particular branch of the sub circuits, taking advantage of the ability to neglect flow source variation and treat flow sources with superposition theorem.

We derive the volume fractions of inlet substances 1 and 2 in the outlet solution by first expressing it in terms of constant branch flow-rates, where Q_N is the flow rate through the Nth circuit branch (Fig. 3).

$$\chi_1 = \frac{Q_1}{Q_1 + Q_2} \quad \text{(S1a)}$$

$$\chi_2 = \frac{Q_2}{Q_1 + Q_2} \quad \text{(S1b)}$$

The flow rates themselves are derived using the fluidic analogy to Ohm's Law, the Hagen-Poiseuille Equation ($\Delta P = QR$), valid in conditions of low Reynold's Number and laminar flow. Here, $P_0 = 0$ to represent atmospheric conditions.

$$Q_1 = \frac{P_0 - P_x}{R_1} = -\frac{P_x}{R_1} \quad \text{(S2a)}$$

$$Q_2 = \frac{P_0 - P_x}{R_2} = -\frac{P_x}{R_2} \quad \text{(S2a)}$$

By inserting S. Eq. 2a,b into S. Eq. 1a,b respectively, the flow-rate independent rules for mixing are simply derived and given by the following:

$$\chi_1 = \frac{R_2}{R_1 + R_2} \tag{S3a}$$

$$\chi_2 = \frac{R_1}{R_1 + R_2} \tag{S3b}$$

Supplemental Note 2

Analysis of 3-inlet 1-output Fork Circuit Topology

Following the analysis in Supplementary Note 1, a 3-input Fork topology follows a similar paradigm (Fig. 4). First, the volume fraction of each inlet substance is described in terms of branch flow rates explicitly.

$$\chi_1 = \frac{Q_1}{Q_1 + Q_2 + Q_3} \tag{S4a}$$

$$\chi_2 = \frac{Q_2}{Q_1 + Q_2 + Q_3} \tag{S4b}$$

$$\chi_3 = \frac{Q_3}{Q_1 + Q_2 + Q_3} \tag{S4c}$$

Next, the Hagen-Poiseuille Law is applied to each branch in order to describe each flow rate in terms of its respective driving pressure and resistance.

$$Q_1 = \frac{P_0 - P_x}{R_1} = -\frac{P_x}{R_1} \tag{S5a}$$

$$Q_2 = \frac{P_0 - P_x}{R_2} = -\frac{P_x}{R_2} \tag{S5b}$$

$$Q_3 = \frac{P_0 - P_x}{R_3} = -\frac{P_x}{R_3} \tag{S5c}$$

Lastly, the flow rates in S. Eq. 5a-c are used to reduce the expression in S. Eq. 4a-c such that the following determined volume fraction is shown to be source flow rate invariant, as in the case 2-1 Fork circuit.

$$\chi_1 = \frac{R_1}{R_1 + R_2 + R_3} \quad \text{(S6a)}$$

$$\chi_2 = \frac{R_2}{R_1 + R_2 + R_3} \quad \text{(S6b)}$$

$$\chi_3 = \frac{R_3}{R_1 + R_2 + R_3} \quad \text{(S6c)}$$

Supplementary Note 3

Analysis of 3-inlet 1-output Ladder Circuit Topology

Following the analysis in Supplementary Note 1, a 3-input Ladder topology follows a similar paradigm (Fig. 5). First, the volume fraction of each inlet substance is described in terms of branch flow rates explicitly.

$$\chi_1 = \frac{Q_1}{Q_1 + Q_2 + Q_3} \quad \text{(S7a)}$$

$$\chi_2 = \frac{Q_2}{Q_1 + Q_2 + Q_3} \quad \text{(S7b)}$$

$$\chi_3 = \frac{Q_3}{Q_1 + Q_2 + Q_3} \quad \text{(S7c)}$$

These expressions are reduced by the Hagen-Poiseuille law, such that:

$$\chi_1 = \frac{-\frac{P_2}{R_1}}{-\frac{P_2}{R_1} - \frac{P_2}{R_2} - \frac{P_3}{R_3}} = \frac{\frac{1}{R_1}}{\frac{1}{R_1} + \frac{1}{R_2} + \frac{\alpha}{R_3}} \quad \text{(S8a)}$$

$$\chi_2 = \frac{-\frac{P_2}{R_2}}{-\frac{P_2}{R_1} - \frac{P_2}{R_2} - \frac{P_3}{R_3}} = \frac{\frac{1}{R_2}}{\frac{1}{R_1} + \frac{1}{R_2} + \frac{\alpha}{R_3}} \quad \text{(S8b)}$$

$$\chi_3 = \frac{-\frac{P_3}{R_3}}{-\frac{P_2}{R_1} - \frac{P_2}{R_2} - \frac{P_3}{R_3}} = \frac{\frac{\alpha}{R_3}}{\frac{1}{R_1} + \frac{1}{R_2} + \frac{\alpha}{R_3}} \quad \text{(S8c)}$$

Where $\alpha = \frac{P_3}{P_2}$. Then through nodal analysis, we are able to find the relative pressures in the circuit. Two equations can be used to describe the conservation of flow in the node nearest to the outlet:

$$\frac{P_2 - P_3}{R_M} - \frac{P_3}{R_3} = Q \quad \text{(S9a)}$$

$$-\frac{P_2}{R_1} - \frac{P_2}{R_2} - \frac{P_3}{R_3} = Q \quad \text{(S9b)}$$

Such that,

$$\alpha = 1 + R_M\left(\frac{1}{R_1} + \frac{1}{R_2}\right) \quad \text{(S10)}$$

Giving a final expression for the volumetric mixing ratio for each branch:

$$\chi_1 = \frac{R_2R_3}{R_2R_3 + R_1R_3 + R_1R_2 + R_M(R_1 + R_2)} \quad \text{(S11a)}$$

$$\chi_2 = \frac{R_1R_3}{R_2R_3 + R_1R_3 + R_1R_2 + R_M(R_1 + R_2)} \quad \text{(S11b)}$$

$$\chi_3 = \frac{R_1R_2 + R_M(R_1 + R_2)}{R_2R_3 + R_1R_3 + R_1R_2 + R_M(R_1 + R_2)} \quad \text{(S11c)}$$

Supplementary Note 4

Experimental Determination of 2-1 Fork Operation

Consider a small volume, dV_3, of concentration c_3 at the outlet resulting form the mixing of two small volumes having run through branch 1 and 2 of the circuit.

$$c_3 dV_3 = c_1 dV_1 + c_2 dV_2 \qquad \text{(S12)}$$

Which can be rewritten in terms of continuous flow, following $Q = \frac{dV}{dt}$,

$$c_3 Q_3 = c_1 Q_1 + c_2 Q_2 \qquad \text{(S13)}$$

From superposition theorem, we treat the flow rates as current through each branch, and proceed to divide both sides of S. Eq. 13 by the total flow rate, giving:

$$c_3 = c_1 \chi_1 + c_2 \chi_2 \qquad \text{(S14)}$$

Assuming that the concentration of solute in branch 2 is set to zero and the stock NaCl solution is to run through branch 1, the operation of the circuit can be verified using the measured values of output and stock NaCl solutions simply, such that:

$$\chi_1 = \frac{c_3}{c_1} = \frac{c_{output}}{c_{stock}} \qquad \text{(S15)}$$

Supplementary Note 5

Experimental Determination of 3-1 Fork Operation

We follow the same methodology as in Note S4 to experimentally determine the volume fraction NaCl through branch 1.

$$c_4 Q_4 = c_1 Q_1 + c_2 Q_2 + c_3 Q_3 \tag{S16}$$

$$Q_4 = Q_1 + Q_2 + Q_3 \tag{S17}$$

$$c_4 = c_1 \chi_1 + c_2 \chi_2 + c_3 \chi_3 \tag{S18}$$

$$c_2 = c_3 = 0 \tag{S19}$$

$$\chi_1 = \frac{c_4}{c_1} = \frac{c_{output}}{c_{stock}} \tag{S20}$$

Supplementary Note 6

Experimental Determination of 3-1 Ladder Operation

Again, we reduce the volume fraction, χ, to a ratio of output concentration to stock concentration.

$$c_4 Q_4 = c_1 Q_1 + c_2 Q_2 + c_3 Q_3 \quad \text{(S21)}$$

$$Q_4 = Q_1 + Q_2 + Q_3 \quad \text{(S22)}$$

$$c_4 = c_1 \chi_1 + c_2 \chi_2 + c_3 \chi_3 \quad \text{(S23)}$$

$$c_2 = c_3 = 0 \quad \text{(S24)}$$

$$\chi_1 = \frac{c_4}{c_1} = \frac{C_{Output}}{C_{Stock}} \quad \text{(S25)}$$
